# A prospective investigation of dietary patterns and internalizing and externalizing mental health problems in adolescents

**DOI:** 10.1002/fsn3.355

**Published:** 2016-03-10

**Authors:** Georgina S. A. Trapp, Karina L. Allen, Lucinda J. Black, Gina L. Ambrosini, Peter Jacoby, Susan Byrne, Karen E. Martin, Wendy H. Oddy

**Affiliations:** ^1^ Telethon Kids InstituteThe University of Western AustraliaPerthAustralia; ^2^ School of Population HealthThe University of Western AustraliaPerthAustralia; ^3^ School of PsychologyThe University of Western AustraliaPerthAustralia; ^4^ Institute of Psychiatry, Psychology and NeuroscienceKing's College LondonUnited Kingdom; ^5^ Maudsley HospitalSouth London and Maudsley NHS Foundation TrustLondonUnited Kingdom; ^6^ School of Public HealthCurtin UniversityPerthAustralia

**Keywords:** Adolescence, diet, externalizing behaviors, mental health, nutrition, raine Study

## Abstract

Investigating protective and risk factors that influence mental health in young people is a high priority. While previous cross‐sectional studies have reported associations between diet and mental health among adolescents, few prospective studies exist. The aim of this study was to examine prospective relationships between dietary patterns and mental health among adolescents participating in the Western Australian Pregnancy Cohort (Raine) Study. Self‐report questionnaires were used to assess indicators of mental health (Youth Self‐Report externalizing/internalizing T‐scores) and Western and Healthy dietary patterns (identified using factor analysis) at 14 (2003–2005) and 17 years (2006–2008). Multivariate linear and logistic regression were used to assess relationships between dietary patterns and mental health. Complete data were available for 746 adolescents. In females only, the Western dietary pattern z–score at 14 years was positively associated with greater externalizing behaviors at 17 years (*β *= 1.91; 95% CI: 0.04, 3.78) and a greater odds of having clinically concerning externalizing behaviors at 17 years (OR = 1.90; 95% CI: 1.06, 3.41). No other statistically significant associations were observed. Overall our findings only lend partial support to a link between diet and mental health. We found it to be specific to females consuming a Western dietary pattern and to externalizing behaviors. Future research on dietary patterns and mental health needs to consider possible sex differences and distinguish between different mental health outcomes as well as between healthy and unhealthy dietary patterns.

## Introduction

Mental health disorders account for nearly a quarter of the world's disability burden (Vos et al. 2013). These disorders include both internalizing problems, such as depression and anxiety, and externalizing problems, such as conduct disorders and attention deficit hyperactivity disorder and may be viewed as occurring on a continuum from normal behavior to problems that warrant a clinical diagnosis. The majority of mental health problems first manifest before adulthood, with marked increases in prevalence between the ages of 16 and 24 years (Jones 2013). Moreover, there is some evidence that the prevalence of mental health problems among young people is increasing with recent estimates suggesting that more than one in four young adults meet the criteria for at least one mental health disorder (Twenge et al. 2010). Adolescence represents a critical period of biological change, rapid growth, and developmental potential, and mental illness during this stage of life may have significant implications for academic success, substance use, and social relationships, both during adolescence and later in life (Fergusson and Woodward 2002). Mental health problems that develop in adolescence also tend to track into adulthood (Hofstra et al. 2001). Therefore, investigating protective and risk factors that influence mental health in young people is a high priority.

Adolescence is often marked by a change in food intake patterns, including missing meals, snacking frequently, and consuming large amounts of energy dense yet nutritionally poor foods (Savige et al. 2012). Poor nutrition has the potential to affect many aspects of brain functioning (Bodnar and Wisner 2005) and there is evidence to suggest a relationship between poor diet and mental health problems. Micronutrient deficiencies, such as omega‐3 fatty acids (Oddy et al. 2011), B‐vitamins (Herbison et al. 2012), magnesium (Jacka et al. 2012), and zinc (Jacka et al. 2012) have been cross‐sectionally associated with an increased risk of depressive mood disorders. Moreover, results from clinical trials suggest that supplementation of specific micronutrients, particularly zinc, may reduce depressive symptoms (Lai et al. 2012). Supplementation of omega‐3 fatty acids may also reduce symptoms of attention‐deficit hyperactivity disorder (ADHD) (Sonuga‐Barke et al. 2013). Investigations into single food factors, such as energy drinks and fast foods, have also been linked to poor mental health (Crawford et al. 2011; Trapp et al. 2013).

A more comprehensive approach than the study of specific nutrients or foods is the examination of diet quality or dietary pattern and mental health. To date, studies which have taken a whole‐diet approach have focused mainly on the relationship between diet and internalizing difficulties, particularly depression and anxiety. A recent systematic review identified 25 studies from nine countries, which examined the association between diet quality or dietary patterns and depression in adults (Quirk et al. 2013). Overall, limited evidence was found to support an association between traditional diets (i.e., Mediterranean diet, Norwegian diet) and depression and mixed results for associations between an “unhealthy” or “healthy” diet and depression, and called for more research in this area. These inconsistencies may relate to the high level of variation in study populations, measurement methods, and covariates included across studies (Quirk et al. 2013). Findings from studies examining the relationship between overall diet and depression in younger age groups also appear to be mixed. For example, an Australian cross‐sectional study reported that 10–14 year olds who consumed a diet with low adherence to the Australian Dietary Guidelines had increased odds of self‐reported symptomatic depression (Jacka et al. 2010a). Similarly, a cross‐sectional study of UK adolescents from varied ethnic and cultural backgrounds found a relationship between higher intakes of unhealthy foods increased scores on the Strengths and Difficulties Questionnaire; SDQ (Jacka et al. 2013). However, Mc Martin and colleagues did not find an association between diet quality and the diagnosis of an internalizing disorder in Canadian children and adolescents (McMartin et al. 2012). Although, children who had greater variety in their diet had significantly lower rates of internalizing disorder in subsequent years, relative to children with little diet variety.

Findings from studies investigating the relationship between diet quality or dietary patterns and anxiety are also mixed and have highlighted some possible gender differences. For example, in Norwegian adults, a “healthy” dietary pattern was associated with reduced anxiety in women and with increased anxiety in men, while a traditional “Norwegian” dietary pattern was associated with increased anxiety in women only and a “Western” dietary pattern was not associated with increased anxiety in either gender (Jacka et al. 2011). In Australian women, a “Traditional” dietary pattern, but not “Modern” or “Western” was associated with reduced anxiety (Jacka et al. 2010b), while in Iranian young adults, a diet high in processed foods was observed to be associated with symptoms of anxiety (Bakhtiyari et al. 2013). At least two cross‐sectional studies investigating overall diet and anxiety have been conducted among younger age groups. Adherence to a “snack” or “animal food” dietary pattern was associated with higher odds of anxiety among Chinese adolescents (Weng et al. 2012), while diet quality, dietary variety, and dietary adequacy were inversely associated with children's feelings of worry, sadness, or unhappiness in Canadian children (McMartin. et al. 2013), with the results being more pronounced in girls than boys.

Few studies have investigated the relationship between overall diet quality or pattern and externalizing behaviors such as conduct problems and aggressive behavior. Recently, we have observed cross‐sectional relationships between a higher score for a ‘Western’ dietary pattern and ADHD (Howard et al. 2011) and higher withdrawal, depressive, delinquent, and aggressive behaviors (Oddy et al. 2009) in the Western Australian Pregnancy Cohort (Raine) Study at 14 years of age. Among younger age groups, a UK study of children participating in the Avon Longitudinal Study of Parents and Children, reported that a ‘junk food’ dietary pattern at age 4.5 years was prospectively associated with increased hyperactivity at age seven, but not with overall behavioral difficulties or conduct and peer problems (Wiles et al. 2007). A German study did not observe a significant relationship between diet quality and conduct problems, hyperactivity or inattention in 9–12 year olds (Kohlboeck et al. 2012). To the best of our knowledge, no previous studies have prospectively investigated the association between overall dietary patterns, including both healthy and Western dietary patterns, and both internalizing and externalizing mental health problems in adolescents.

In reviewing the literature to date, micronutrients, individual foods, diet quality, and dietary pattern scores have all been linked to mental health problems, particularly internalizing (depressive, anxiety) difficulties. For externalizing mental health difficulties, links appear to be stronger with unhealthy dietary patterns than with healthy patterns. Furthermore, some studies have highlighted gender differences in the relationship between overall diet and mental health problems. This highlights the importance of attending to both aspects of dietary quality, healthy and unhealthy, and examining both aspects of mental health, internalizing and externalizing, as well as exploring possible gender differences when conducting research in this area.

The current analysis aims to extend previous cross‐sectional findings in the Raine cohort by examining the prospective relationship between dietary patterns at 14 years and mental health at 17 years in participants. We hypothesized that a diet high in the ‘Western’ dietary pattern would be a prospective predictor of mental health problems, while a diet high in the ‘Healthy’ dietary pattern would be protective against such problems.

## Methods

### Participants

Participants were drawn from the Western Australian Pregnancy Cohort (Raine) Study. Detailed methods regarding the Raine Study have been published previously (Newnham et al. 1993). In brief, 2900 West Australian women were recruited in pregnancy through the public antenatal clinic and local private clinics in Perth (1989–1991) and gave birth to 2868 children. Data were collected from the mothers, partners, and children at 18 and 34 weeks gestation, birth, and 1, 2, 3, 5, 8, 10, 14, and 17 years. This study focuses on participants who provided diet and mental health data during the 14 year (2003–2005) and 17 year (2006–2008) follow‐ups. Data from earlier follow‐ups were used when describing the socio‐demographic characteristics of the participants. Informed consent was obtained from the primary caregiver as well as from the adolescent. The Raine Study protocol was approved by the ethics committees of King Edward Memorial Hospital for Women and Princess Margaret Hospital for Children. Details of cohort attrition have been documented previously (Li et al. 2008; Robinson et al. 2010). Follow‐up rates have been approximately 75–90% of those available for follow‐up, which is comparable to, or better than, similar cohorts (Wolke et al. 2009).

### Assessment of mental health

Indicators of mental health were assessed at the 14 and 17 year follow‐ups using the Youth Self‐Report (YSR), an adolescent self‐report version of the Child Behaviour Checklist for Ages 4–18 (CBCL/4–18). The YSR is a 118‐item empirically validated and reliable measure of emotional and behavioral problems in children and adolescents (Achenbach 1991; Achenbach T. 2001). The YSR yields an externalizing problem score that describes ‘acting out’ behaviors such as conduct problems and aggressive behavior, and an internalizing problem score that describes depressive and anxiety symptoms and withdrawn behavior (Achenbach 1991; Achenbach T. 2001). Standardized T‐scores, normalized separately for boys and girls by age, for externalizing and internalizing problem scales were calculated, with higher scores indicating a higher level of emotional and behavioral problems. YSR T‐scores ≥60 for both the internalizing and externalizing scale were considered clinically concerning (Achenbach 1991).

### Assessment of dietary patterns

Identification of the Western and Healthy dietary patterns at the 14 year follow‐up has been described previously (Ambrosini et al. 2009b). Briefly, the study adolescent, with the assistance of their primary caregiver, completed a validated semiquantitative food frequency questionnaire (FFQ) developed by the Commonwealth Scientific and Industrial Research Organisation (CSIRO) (Ambrosini et al. 2009a). The FFQ assessed dietary intake over the previous year and estimated usual frequency of consumption and serve size information on 212 foods items or dishes, which were subsequently collapsed into 38 food groups according to culinary usage and nutritional properties (Ambrosini et al. 2009b). Factor analysis was then used to reduce the intakes of all food groups into a smaller number of dietary patterns that summarized total dietary intake. Two major dietary patterns were identified. The Healthy pattern comprised higher intakes of whole grains, fruit, vegetables, legumes, and fish, and the Western pattern was characterized by high intakes of take‐away foods, red meats, processed meats, full‐fat dairy products, fried potatoes, refined cereals, cakes and biscuits, confectionary, soft drinks, crisps, sauces, and dressings (Ambrosini et al. 2009b). Each participant received a z‐score for both dietary patterns, indicating the degree to which their reported dietary intake reflected each dietary pattern.

### Potential confounding variables

As dietary misreporting can obscure diet–outcome relationships, the Goldberg method (Poslusna et al. 2009) was used to identify dietary under‐reporters, plausible reporters, or over‐reporters at 14 years of age. This is a widely used method that has been previously applied in this cohort (Oddy et al. 2013). We adjusted for total energy intake (kilojoules) at 14 years to examine whether observed associations between dietary patterns and mental health scores were independent of the adolescent's total energy intake (Willett et al. 1997). At 17 years, a trained research assistant recorded height and weight measurements using standard calibrated equipment. Body mass index (BMI) was calculated as body weight (kg)/height (m)^2^ and treated as a continuous variable. In addition, participants were asked how many times they engaged in physical activity that caused breathlessness or sweating, outside of school hours. Responses were categorized into ≤1/week or less, 1–3 times/week, and 4 +times/week.

We included maternal education at the time of recruitment (completed Year 12 high school, yes/no) and quartiles of annual household income at 17 years as indices of socio‐demographic status. The General Functioning Scale from the McMaster Family Assessment Device was used to assess family functioning at 17 years (Epstein et al. 1983). This scale is reliable and internally consistent (Byles et al. 2004) with lower scores representing poorer and higher scores representing better functioning. Due to the skewness of the raw scores, natural log transformations were applied.

### Statistical analysis

Descriptive statistics were computed for socio‐demographics, lifestyle, dietary intake, and mental health variables. Chi square and independent sample t‐tests were used to identify differences in characteristics between participants who completed and did not complete the follow‐ups and between male and female participants. Multivariate linear regression was used to examine the association between dietary patterns and YSR T‐scores. In addition, we used multivariate logistic regression to examine the association between dietary patterns and clinically concerning YSR T‐scores. All models included z‐scores for both dietary patterns and were adjusted for maternal education at birth, confounders at 14 years: dietary misreporting, YSR T‐scores; and confounders at 17 years: family income, family functioning, physical activity, and BMI. A second model further adjusted for energy intake. Interactions between dietary pattern and gender were examined for each mental health outcome and none were found to be statistically significant (all *P *<* *0.05). However, given the significant gender differences in absolute levels of mental health and dietary variables identified in this sample, analyses were stratified by gender. Analyses were performed using SPSS Version 20 (IBM SPSS Inc., Chicago, IL).

## Results

### Characteristics of participants

A total of 1861 adolescents (84% of those eligible), participated in the 14 year follow‐up and 1754 (81% of those eligible) participated in the 17‐year follow‐up. Participants included in this study were adolescents who provided complete data at both the 14 and 17 year follow‐ups (*n* = 746). The characteristics of these adolescents compared with nonparticipants are presented in Table 1.

**Table 1:** Characteristics of participants included in this study compared with nonparticipants from the original cohort

	Participants(*n* = 746)%	Nonparticipants(*n* = 2122)%
Maternal age at birth (M[SD])	30.0 (5.6)	27.5 (5.9)***
Maternal BMI^a^ (M[SD])	22.1 (3.9)	22.4 (4.4)*
Mother drinking alcohol^1^		
Never	0.4	0.8**
Less than once a week	5.8	4.9
Approximately once a week	9.5	10.1
Several times a week	34.8	27.9
Daily	49.5	56.3
Mother smoking cigarettes^1^		
None	82.2	69.7***
1–5 daily	6.7	9.0
6–10 daily	4.2	8.2
11–15 daily	3.4	6.3
16–20 daily	2.7	4.3
21 or more per day	0.9	2.6
Mother completed secondary school^1^	73.9	70.5
Family income^a^		
< $7,000	3.7	10.4***
$7,000 ‐ $11,999	5.5	10.5
$12,000‐$23,999	21.2	27.8
$24,000‐$35,000	25.9	24.3
$35,001 or more	43.6	27.1
Biological father living at home^a^	92.6	84.6***
Offspring gestational age at birth (weeks) (M[SD])	39 (2)	39 (2)**
Offspring birth weight (kg) (M[SD])	3.34 (0.57)	3.27 (0.64)**
Preterm birth (<37 weeks)	7.1	9.6*
Offspring sex (% male)	49.2	51.3

*P < 0.05, **P < 0.01, ***P < 0.001. BMI: Body Mass Index, M: Mean, SD: Standard Deviation.

a Measured at 18 weeks gestation,

The percentages or means and standard deviations of all measures are presented in Table 2. Compared with males, females had significantly lower Western pattern z‐scores, total energy intakes (kj/day), and physical activity levels and were more likely to misreport dietary intake. Females also had significantly higher family functioning scores, Internalizing T‐scores (at 17 years), Externalizing T‐scores (at 14 and 17 years) and had a greater proportion with clinically concerning YSR T‐scores (at 14 and 17 years).

**Table 2:** Characteristics of participants who provided complete data at the 14 year and 17 year follow‐ups (*n* = 746)

	Males	Females
Socio‐demographics		
Family functioning at 17 years (mean [SD])^a^	1.78 (0.43)	1.84 (0.48)*
Family income at 17 years (%)		
Quartile 1: ≤$50,000	21.0	26.1
Quartile 2: $50,001–$78,000	22.6	22.4
Quartile 3: $78,001–$104,000	22.3	17.7
Quartile 4: >$104,000	34.1	33.8
Lifestyle factors		
Physical activity at 17 years (%)		
<1 day/week	13.1	29.6***
1–3 days/week	54.5	50.9
4 + days/week	32.4	19.5
BMI (kg/m^2^) at 17 years (mean [SD])	22.51 (3.99)	22.84 (4.27)
Mental health		
bYSR Internalizing T‐score (mean [SD])		
14 years	46.78 (9.86)	47.93 (9.21)
17 years	46.66 (10.96)	50.28 (10.31)***
Clinically concerning YSR Internalizing T‐score (%)		
14 years	11.2	8.2
17 years	12.8	17.4
YSR Externalizing T‐score (mean [SD])^b^		
14 years	47.59 (9.57)	51.22 (9.87)***
17 years	49.47 (10.06)	52.18 (10.23)***
Clinically concerning YSR Externalizing T‐score (%)		
14 years	9.5	19.8***
17 years	14.2	22.7**
Dietary intake		
Healthy pattern z score at 14 years (mean [SD])	−0.01 (0.88)	0.05 (0.81)
Western pattern z score at 14 years (mean [SD])	0.07 (0.79)	−0.29 (0.81)***
Energy intake at 14 years (KJ/day (mean [SD])	10,255.2 (2870.0)	8438.4 (2717.6)***
Dietary misreporting at 14 years (%)		
Under‐reporting	11.7	42.5***
Plausible reporting	70.8	54.6
Over‐reporting	17.4	2.9

*P < 0.05, **P < 0.01, ***P < 0.001. YSR: Youth Self‐Report; BMI: Body Mass Index, M: Mean, SD: Standard Deviation.

a Lower scores represent poorer, and higher scores represent better family functioning.

b Higher scores indicate a higher level of emotional and behavioral problems.

### Associations between dietary patterns at 14 years and mental health outcomes at 17 years

No statistically significant associations were observed between Western or Healthy dietary pattern z‐scores at 14 years and externalizing or internalizing behaviors at 17 years after adjustment for mental health at 14 (i.e., corresponding internalizing or externalizing T‐scores), family income at 17, family functioning at 17, physical activity at 17, BMI at 17, and maternal education at birth, other dietary pattern at 14, and dietary misreporting at 14 (Model 1, Tables 3 and 4). However, once total energy intake was added to the model, a one‐Standard Deviation (SD) unit higher z‐score for the Western dietary pattern at 14 years was associated with an average increase of 1.91 in Externalizing T‐score at the 17 year follow‐up (*β *= 1.91; 95% CI: 0.04, 3.78) (Table 3, Model 2). Similarly for females, a one‐SD unit higher z‐score for the Western dietary pattern at 14 years was associated with nearly double the odds of having clinically concerning Externalizing T‐scores at 17 years of age (OR 1.90; 95% CI: 1.06, 3.41) (Table 4, Model 2).

**Table 3:** Adjusted multivariate general linear and logistic regression coefficients for the effect of dietary pattern at 14 years on Youth Self‐Report Externalizing and Internalizing T‐scores at 17 years (*n* = 746)

	YSR Internalizing T‐scores				YSR Externalizing T‐scores			
	Model 1^a^		Model 2^b^		Model 1^a^		Model 2^b^	
	*β* (95%CI)	*P*	*β* (95%CI)	*P*	*β* (95%CI)	*P*	*β* (95%CI)	***P***
Males								
Healthy dietary pattern	−0.69 (−1.87, 0.48)	0.248	−0.60 (−1.96, 0.76)	0.390	−0.25 (−1.24, 0.72)	0.608	−0.50 (−1.53, 0.54)	0.348
Western dietary pattern	−0.05 (−1.66, 1.57)	0.955	−0.35 (−2.28, 1.58)	0.721	0.60 (−0.75, 1.95)	0.384	−0.02 (−1.63, 1.59)	0.979
Females								
Healthy dietary pattern	−0.03 (−1.16, 1.09)	0.955	−0.14 (−1.57, 1.29)	0.848	0.07 (−1.02, 1.17)	0.895	0.59 (−0.62, 1.79)	0.338
Western dietary pattern	−0.43 (−1.91, 1.05)	0.566	−0.83 (−3.05, 1.39)	0.463	0.70 (−0.75, 2.15)	0.343	1.91 (0.04, 3.78)	0.045

YSR: Youth Self‐Report.

a Adjusted for mental health at 14 (i.e., corresponding internalizing or externalizing T‐scores), family income at 17, family functioning at 17, physical activity at 17, Body Mass Index at 17 and maternal education at birth, other dietary pattern at 14, and dietary misreporting at 14.

b Further adjusted for total energy intake.

**Table 4:** Adjusted multivariate general linear and logistic regression coefficients for the effect of dietary pattern at 14 years on clinically concerning Youth Self‐Report Externalizing and Internalizing T‐scores at 17 years (*n* = 746)

	Clinically concerning YSR Internalizing T‐scores				Clinically concerning YSR Externalizing T‐scores			
	Model 1^a^		Model 2^b^		Model 1^a^		Model 2^b^	
	*β* (95%CI)	*P*	*β* (95%CI)	*P*	*β* (95%CI)	*P*	*β* (95%CI)	*P*
Males								
Healthy dietary pattern	0.76 (0.49, 1.17)	0.212	0.65 (0.41, 1.03)	0.066	1.04 (0.69, 1.54)	0.864	0.93 (0.61, 1.40)	0.720
Western dietary pattern	0.89 (0.52, 1.54)	0.892	0.61 (0.32, 1.17)	0.136	1.53 (0.88, 2.66)	0.131	1.11 (0.59, 2.10)	0.740
Females								
Healthy dietary pattern	0.99 (0.69, 1.44)	0.994	0.92 (0.61, 1.38)	0.684	0.89 (0.63, 1.27)	0.517	0.99 (0.68, 1.46)	0.986
Western dietary pattern	0.91 (0.56, 1.47)	0.704	1.05 (0.98, 1.12)	0.376	1.43 (0.92, 2.23)	0.110	1.90 (1.06, 3.41)	0.030

YSR: Youth Self‐Report.

a Adjusted for mental health at 14 (i.e., corresponding internalizing or externalizing T‐scores), family income at 17, family functioning at 17, physical activity at 17, Body Mass Index at 17 and maternal education at birth, other dietary pattern at 14, and dietary misreporting at 14.

b Further adjusted for total energy intake.

## Discussion

This study presents unique prospective data on associations between dietary patterns and mental health in adolescents. We hypothesized that a diet high in the ‘Western’ dietary pattern would be a prospective predictor of mental health problems, while a diet high in the ‘Healthy’ dietary pattern would be protective against such problems. Of these two hypotheses, only the first received partial support. A higher score for a Western dietary pattern at 14 years was associated with a significant increase in externalizing behaviors and a greater odds of having clinically concerning externalizing behaviors at 17, but only in females after full adjustment of confounders including total energy intake.

This study supports and extends our previous cross‐sectional findings, which linked a Western dietary pattern, but not a Healthy dietary pattern, to greater externalizing behaviors (Oddy et al. 2009) as well as ADHD (Howard et al. 2011) at 14 years of age in both boys and girls. Thus, for externalizing mental health difficulties, we have found stronger links with an unhealthy dietary pattern than with a healthy pattern. Our findings are consistent with a German cross‐sectional study on 10 year olds, which did not find evidence of a relationship between diet quality (as measured by a German optimized diet score), conduct problems, and hyperactivity (Kohlboeck et al. 2012), and a UK study which reported an association between a ‘junk food’ dietary pattern at age 4.5 and increased hyperactivity at age seven (Wiles et al. 2007). Overall, our findings suggest that discouraging high intakes of takeaway foods, confectionary, processed meat, refined grains, and soft drinks may be a useful strategy in the prevention of externalizing mental health disorders in young people, particularly females.

Why we did not observe an association between a Western dietary pattern and externalizing problems in males is unclear. It may relate to females in this sample having significantly greater Externalizing T‐scores and clinically concerning Externalizing T‐scores at 14 and 17 years. This is consistent with other studies showing higher rates of many mental health disorders in females compared to males (Essau et al. 2010). It is also plausible that hormonal influences may account for these gender differences, since possible mediating factors between diet and mental health, such as inflammation and oxidative stress, are influenced by gonadal hormones (Kher et al. 2005). Given that other studies have also observed gender differences in the diet–mental health relationship, it is important that gender differences are examined when conducting further research in this area.

Our observed relationship between a Western dietary pattern at 14 years and externalizing behaviors at 17 years in females was only significant after adjustment of confounders including total energy intake. This suggests that in females, it is the amount of unhealthy food as a proportion of overall energy intake that is relevant, not the absolute amount. Similarly, and as noted in the systematic review by Quirk and colleagues (Quirk et al. 2013), there have been other studies where relationships between dietary patterns and mental health were stronger after adjustment for total energy intake (Jacka et al. 2010b, 2011). Thus, future studies investigating the relationship between overall diet and mental health difficulties should take this into account and present models with and without adjustment for total energy intake.

We did not find support for a relationship between a Healthy dietary pattern at 14 years and internalizing problem scores age 17. This is in contrast to several adult studies (Quirk et al. 2013) and one study on 10–14 year olds which found evidence for a relationship between a healthy diet and reduced likelihood of depression. Our results are consistent, however, with several adult and child studies which found no evidence for a relationship between depression and a healthy diet and with the mixed literature on possible links between overall diet and anxiety (McMartin et al. 2012; Quirk et al. 2013). This inconsistency in the literature may relate, at least in part, to variation in measurement methods for dietary patterns. For example, recent findings from the Nurses’ Health Study showed that while associations between dietary patterns and depression were originally not detected (Chocano‐Bedoya et al. 2013), examining the dietary components using Reduced‐Rank Regression (RRR) instead of factor analysis (Principal Components Analysis [PCA]), according to the relationship of dietary variables to inflammation, resulted in statistically significant associations between dietary patterns and depression over time (Lopez‐Garcia et al. 2004). For the current research, our prospective analysis follows on from previously published cross‐sectional analysis of dietary patterns derived using factor analysis, which has been commonly applied in the nutritional epidemiology literature (Newby and Tucker 2004) and been shown to be a reliable method for identifying dietary patterns (Khani et al. 2004). Despite this, it is possible that alternative methodologies, including RRR, would yield different results and this should be considered in future research. Future studies may also benefit from taking into account more sensitive measures that better associate with mental health, such as inflammation. Overall, additional research investigating the relationship between overall diet and internalizing problems is needed.

Possible mechanisms that may underpin the relationship between diet and mental health include inflammation (Zunszain et al. 2013), immune system dysfunction (Pasco et al. 2010), oxidative stress (Ng et al. 2008), and biochemistry (Shimizu et al. 2003). Given that diet is a modifiable risk factor that has been linked to mental health problems, it is important that more research in this relatively new and growing area of research is continued. Future research on overall diet and mental health should consider possible sex differences and distinguish between different mental health outcomes as well as between healthy and unhealthy dietary patterns because our findings suggest that it may only be in specific areas that associations are evident. Future research could also examine changes in diet over time to determine if change in diet leads to subsequent changes in mental health. Furthermore, a better understanding of the period of time over which dietary exposures have an effect on mental health is needed. While this study highlights the importance of diet and its potential role in modifying mental health in adolescence, we only examined a time period of 3 years and thus it is possible that shorter or longer time periods may also be important.

### Study limitations and strengths

The strengths of our study include a prospective study design, a large population‐based cohort, a validated measure of mental health, the use of dietary pattern analysis which summarizes the total diet, and adjustment for a wide range of relevant confounding factors. Although we cannot rule out the possibility that dietary and mental health data were subject to self‐report biases, we did adjust for dietary misreporting. Use of complete case analysis restricted the number of participants in our final models to 747. Survey completion was not as high at the 17 year follow‐up compared with the 14 year follow‐up. This is likely due to most participants being in their final year of high school and completing their exams. For example, only 993 participants completed the FFQ at the 17 year follow‐up, compared with 1613 at the 14 year follow‐up. Care should be taken when generalizing these results to the wider community as participants in this study were more likely to be socioeconomically advantaged relative to participants lost to follow‐up. While we did adjust for a wide array of confounders, there are likely to have been other factors (e.g., social and policy factors) not controlled for in our analyses that may have influenced the relationship between diet and mental health. Finally, it is possible that the relationship between diet and mental health is bidirectional, and causality cannot be established in the current analysis. That is, adolescents experiencing emotional distress may turn to foods that are high in fat, sugar, and salt as a coping mechanism for psychological symptoms or as a result of appetite change.

## Conclusion

Overall, our findings lend partial support to a link between overall diet and mental health. We found it to be specific to females consuming a Western dietary pattern and to externalizing behaviors. Future research on dietary patterns and mental health needs to consider possible sex differences and distinguish between different mental health outcomes as well as between healthy and unhealthy dietary patterns because our findings suggest that it may only be in specific areas that associations are evident. It is also important that future research seeks to elucidate biological pathways that may mediate the relationships between diet and mental health, as well as intervention studies that seek to provide evidence of causality.

## Conflict Interests

None declared.
